# Supplementary material for: Heparin-based hydrogel scaffolding alters the transcriptomic profile and increases the chemoresistance of MDA-MB-231 triple-negative breast cancer cells
Source: Biomater Sci. 2020 Feb 13;8(10):2786–96. doi: 10.1039/c9bm01481k (PMC7497406; doi:10.1039/c9bm01481k)
Supplement: Supplementary file 2 [file BM-008-C9BM01481K-s002.zip › Supplementary File 4/HP-BhydrogelvControl/Pathways/my_analysis.Gsea.1545201079907/HALLMARK_BILE_ACID_METABOLISM.html]

Details for gene set HALLMARK\_BILE\_ACID\_METABOLISM[GSEA]

|  || Dataset | expr.class.cls#HYD\_versus\_CONTROL.class.cls#HYD\_versus\_CONTROL\_repos |
| Phenotype | class.cls#HYD\_versus\_CONTROL\_repos |
| Upregulated in class | CONTROL |
| GeneSet | HALLMARK\_BILE\_ACID\_METABOLISM |
| Enrichment Score (ES) | -0.3123857 |
| Normalized Enrichment Score (NES) | -1.3169878 |
| Nominal p-value | 0.047445256 |
| FDR q-value | 0.06908405 |
| FWER p-Value | 0.454 |
Table: GSEA Results Summary

  

Fig 1: Enrichment plot: HALLMARK\_BILE\_ACID\_METABOLISM      
 Profile of the Running ES Score & Positions of GeneSet Members on the Rank Ordered List

  

| PROBE | DESCRIPTION (from dataset) | GENE SYMBOL | GENE\_TITLE | RANK IN GENE LIST | RANK METRIC SCORE | RUNNING ES | CORE ENRICHMENT || 1 | MLYCD | na |  |  | 231 | 2.846 | 0.0353 | No |
| 2 | PAOX | na |  |  | 1338 | 1.661 | 0.0051 | No |
| 3 | ABCA2 | na |  |  | 1340 | 1.660 | 0.0327 | No |
| 4 | LIPE | na |  |  | 1405 | 1.627 | 0.0564 | No |
| 5 | PEX6 | na |  |  | 1559 | 1.547 | 0.0741 | No |
| 6 | BCAR3 | na |  |  | 2209 | 1.272 | 0.0614 | No |
| 7 | RXRA | na |  |  | 3265 | 0.972 | 0.0224 | No |
| 8 | EFHC1 | na |  |  | 3282 | 0.968 | 0.0376 | No |
| 9 | FDXR | na |  |  | 3632 | 0.886 | 0.0341 | No |
| 10 | PFKM | na |  |  | 3663 | 0.879 | 0.0472 | No |
| 11 | HACL1 | na |  |  | 3848 | 0.842 | 0.0516 | No |
| 12 | TFCP2L1 | na |  |  | 4036 | 0.805 | 0.0552 | No |
| 13 | PEX16 | na |  |  | 4680 | 0.680 | 0.0329 | No |
| 14 | NR3C2 | na |  |  | 5098 | 0.613 | 0.0213 | No |
| 15 | ABCD1 | na |  |  | 5174 | 0.604 | 0.0274 | No |
| 16 | GNMT | na |  |  | 5481 | 0.555 | 0.0206 | No |
| 17 | SLC23A1 | na |  |  | 5523 | 0.549 | 0.0276 | No |
| 18 | ABCA5 | na |  |  | 5661 | 0.531 | 0.0293 | No |
| 19 | GSTK1 | na |  |  | 5693 | 0.526 | 0.0364 | No |
| 20 | SLC22A18 | na |  |  | 5759 | 0.518 | 0.0416 | No |
| 21 | BMP6 | na |  |  | 6580 | 0.405 | 0.0055 | No |
| 22 | ABCG4 | na |  |  | 6690 | 0.394 | 0.0064 | No |
| 23 | PIPOX | na |  |  | 7325 | 0.323 | -0.0214 | No |
| 24 | SLC23A2 | na |  |  | 7458 | 0.312 | -0.0231 | No |
| 25 | ABCA3 | na |  |  | 7496 | 0.308 | -0.0199 | No |
| 26 | PEX11G | na |  |  | 7875 | 0.264 | -0.0353 | No |
| 27 | PEX26 | na |  |  | 7894 | 0.262 | -0.0319 | No |
| 28 | CROT | na |  |  | 8403 | 0.207 | -0.0550 | No |
| 29 | ATXN1 | na |  |  | 8586 | 0.195 | -0.0613 | No |
| 30 | SLC27A5 | na |  |  | 8662 | 0.185 | -0.0622 | No |
| 31 | CYP46A1 | na |  |  | 8767 | 0.171 | -0.0648 | No |
| 32 | ABCA6 | na |  |  | 8809 | 0.166 | -0.0641 | No |
| 33 | FADS2 | na |  |  | 9328 | 0.112 | -0.0894 | No |
| 34 | HSD17B6 | na |  |  | 10042 | 0.036 | -0.1261 | No |
| 35 | CYP27A1 | na |  |  | 10067 | 0.034 | -0.1267 | No |
| 36 | ABCA1 | na |  |  | 10121 | 0.026 | -0.1291 | No |
| 37 | RETSAT | na |  |  | 10420 | 0.005 | -0.1446 | No |
| 38 | GCLM | na |  |  | 11055 | -0.073 | -0.1765 | No |
| 39 | FADS1 | na |  |  | 11338 | -0.094 | -0.1897 | No |
| 40 | NEDD4 | na |  |  | 11486 | -0.108 | -0.1956 | No |
| 41 | HSD3B7 | na |  |  | 11566 | -0.122 | -0.1977 | No |
| 42 | OPTN | na |  |  | 11715 | -0.140 | -0.2031 | No |
| 43 | DHCR24 | na |  |  | 11725 | -0.142 | -0.2012 | No |
| 44 | DIO2 | na |  |  | 12462 | -0.222 | -0.2360 | No |
| 45 | PXMP2 | na |  |  | 13357 | -0.330 | -0.2773 | No |
| 46 | ACSL5 | na |  |  | 13564 | -0.364 | -0.2820 | No |
| 47 | NPC1 | na |  |  | 13743 | -0.394 | -0.2848 | No |
| 48 | GNPAT | na |  |  | 13747 | -0.395 | -0.2784 | No |
| 49 | SLCO1A2 | na |  |  | 13958 | -0.415 | -0.2824 | No |
| 50 | NR1I2 | na |  |  | 13986 | -0.415 | -0.2769 | No |
| 51 | PEX7 | na |  |  | 14192 | -0.440 | -0.2803 | No |
| 52 | IDH2 | na |  |  | 14279 | -0.459 | -0.2772 | No |
| 53 | ACSL1 | na |  |  | 14604 | -0.507 | -0.2857 | No |
| 54 | ABCD3 | na |  |  | 14612 | -0.508 | -0.2776 | No |
| 55 | SLC35B2 | na |  |  | 15200 | -0.595 | -0.2984 | No |
| 56 | PEX11A | na |  |  | 15218 | -0.600 | -0.2893 | No |
| 57 | PEX12 | na |  |  | 15660 | -0.678 | -0.3011 | Yes |
| 58 | ISOC1 | na |  |  | 15866 | -0.717 | -0.2999 | Yes |
| 59 | PEX1 | na |  |  | 15880 | -0.720 | -0.2886 | Yes |
| 60 | SLC29A1 | na |  |  | 16048 | -0.759 | -0.2847 | Yes |
| 61 | LONP2 | na |  |  | 16328 | -0.816 | -0.2857 | Yes |
| 62 | IDI1 | na |  |  | 16426 | -0.838 | -0.2768 | Yes |
| 63 | SCP2 | na |  |  | 16821 | -0.932 | -0.2819 | Yes |
| 64 | CYP39A1 | na |  |  | 17115 | -1.019 | -0.2803 | Yes |
| 65 | APOA1 | na |  |  | 17228 | -1.058 | -0.2685 | Yes |
| 66 | PRDX5 | na |  |  | 17276 | -1.076 | -0.2531 | Yes |
| 67 | PHYH | na |  |  | 17292 | -1.081 | -0.2359 | Yes |
| 68 | PEX13 | na |  |  | 17316 | -1.089 | -0.2189 | Yes |
| 69 | PECR | na |  |  | 17342 | -1.099 | -0.2020 | Yes |
| 70 | AMACR | na |  |  | 17354 | -1.102 | -0.1842 | Yes |
| 71 | HSD17B4 | na |  |  | 17589 | -1.191 | -0.1766 | Yes |
| 72 | AR | na |  |  | 17770 | -1.274 | -0.1648 | Yes |
| 73 | PEX19 | na |  |  | 17905 | -1.335 | -0.1496 | Yes |
| 74 | ALDH9A1 | na |  |  | 18095 | -1.437 | -0.1356 | Yes |
| 75 | IDH1 | na |  |  | 18161 | -1.475 | -0.1144 | Yes |
| 76 | PNPLA8 | na |  |  | 18625 | -1.812 | -0.1085 | Yes |
| 77 | NUDT12 | na |  |  | 18652 | -1.849 | -0.0791 | Yes |
| 78 | HSD17B11 | na |  |  | 18720 | -1.913 | -0.0507 | Yes |
| 79 | SOD1 | na |  |  | 18799 | -2.015 | -0.0213 | Yes |
| 80 | CAT | na |  |  | 19052 | -2.529 | 0.0076 | Yes |
Table: GSEA details [plain text format]

  

Fig 2: HALLMARK\_BILE\_ACID\_METABOLISM      
 Blue-Pink O' Gram in the Space of the Analyzed GeneSet

  

Fig 3: HALLMARK\_BILE\_ACID\_METABOLISM: Random ES distribution      
 Gene set null distribution of ES for **HALLMARK\_BILE\_ACID\_METABOLISM**
